# The stealth *Francisella*: The first fatality caused by *Francisella novicida* bacteraemia in Malaysia

**DOI:** 10.1016/j.idcr.2025.e02387

**Published:** 2025-10-09

**Authors:** Habib Esa, Guan Han Lin, Woo Yen Yen, Khayri Kamel, Oon Chin Liang, Revathy Arushothy, Shirley Yi Fen Hii, Nur Asyura Nor Amdan, Letchumy Sundaraj, Nur Fatihah Abu Bakar, Nazirah Samsudin, Rohaidah Hashim

**Affiliations:** a Bacteriology Unit, Infectious Disease Research Centre, Institute for Medical Research, National Institutes of Health (NIH), Shah Alam, Selangor 40170, Malaysia; b Medicine Department, Seberang Jaya Hospital, Seberang Jaya, Perai, Pulau Pinang 13700, Malaysia; c Microbiology Department, Seberang Jaya Hospital, Seberang Jaya, Perai, Pulau Pinang 13700, Malaysia; d Virology Unit, Infectious Disease Research Centre, Institute for Medical Research, NIH, Shah Alam, Selangor 40170, Malaysia

**Keywords:** *Francisella novicida*, Bacteraemia, Next generation sequencing

## Abstract

*Francisella novicida* is a rare opportunistic pathogen that is closely related to *Francisella tularensis* subspecies tularensis and subspecies holartica. However, it does not cause tularaemia and is not transmitted to humans through arthropod bites. This case is the first time this bacterium has been isolated from a patient in Malaysia. The patient was a 69-year-old woman with hypertension and dyslipidaemia, who unfortunately passed away due to the infection. She presented with infective gastroenteritis and deteriorated into full blown Gram-negative bacteraemia. Despite treatment with meropenem, she succumbed to the infection. Identification attempts using conventional and mass spectrometry method failed. The microbiological confirmation relied on next generation sequencing. This case highlights the challenges faced on bacterial identification, possible empirical treatment options for unidentified gram-negative bacteraemia and implication of rare bacterial infection.

## Introduction

Tularaemia is a potentially life-threatening zoonotic infection that spreads through tick bites. It is caused by *Francisella tularensis* subspecies *holartica* (FH) and *Francisella tularensis* subspecies *tularensis* (FT). Despite being closely related with average nucleotide identify of > 98 % to related to FH and FT, *Francisella novicida* (FN) does not cause tularaemia [1]. Currently, FN is considered as a separate species, because of significant differences in the genomic, clinical presentation, virulence and ecological properties [1], [2]. Although being less virulent, FN can cause mortality and significant morbidity especially in immunocompromised hosts [3], [4], [5], [6], [7], [8], [9]. Even though the number of reported cases has risen over the last ten years, human infections are still rare worldwide. There have been no previous reports of FN bacteraemia in humans in Malaysia. We report the first fatal case of FN bacteraemia in a 69-year-old woman in Malaysia, highlighting the challenges in identifying the bacteria with standard diagnostic tools in Malaysian public health hospitals.

## Case report

A 69-year-old Indian lady presented with fever, diarrhoea and vomiting for five days admitted to the medical ward of Seberang Jaya Hospital, Penang on 12th August 2023. She had underlying diabetes, hypertension and dyslipidaemia. She worked as a cleaner in an office located in a factory in Seberang Jaya, Penang, Malaysia. Four days prior to the admission, she went to a private practitioner and was treated for acute gastroenteritis. However, her symptoms were not improving thus she sought further treatment in the hospital. She was given intravenous ampicillin-sulbactam. Blood tests including cultures were taken as part of the workup (Table 1). Both her white blood cell count and inflammatory markers were elevated but her vital signs were stable apart from mild grade fever. On day two of admission, she had spikes of high-grade fever and further examination revealed crepitation on the right middle and upper zones of lungs. Chest X-ray revealed consolidation in the right middle to upper zones of lungs indicative of pneumonia. Community acquired pneumonia was diagnosed. Oral azithromycin 500 mg daily was added to her treatment regimen as empirical for atypical infection However, her condition worsened as she became tachypnoeic, tachycardic and hypotensive on day three of admission. She was intubated for securing the airway and admitted to the intensive care unit for critical care. Upon review by the infectious disease physician, melioidosis was suspected due to its classical presentation and high prevalence in Southeast Asia. Thus, IV ceftazidime was started.

**Table 1:** Summary of investigation results from day 2 to day 8 of admission.

**Fig. 1:**
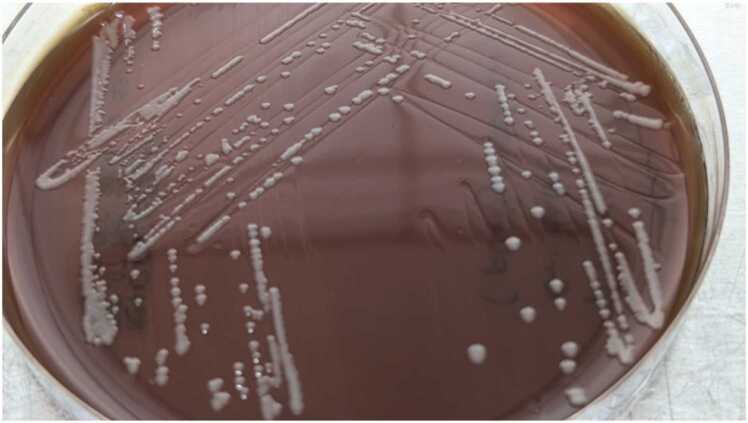
Colonies on a sheep blood agar after 72 h of incubation under aerobic incubation at 37 ± 2 °C. The colonies were ∼ 1 mm in diameter with greyish, smooth round, and non-haemolytic.

Concurrently, her blood culture was positive for gram-negative tiny coccobacilli. The gram-negative coccobacilli grew slowly on blood agar after 48 h of incubation as tiny and translucent colonies. After 72 h of incubation under aerobic condition at 37 ± 2 °C, the colonies appear as greyish, smooth-round, and non-haemolytic (Fig. 1). Biochemical reactions were tested negative for both oxidase and urease but weakly positive for catalase test. Both Vitek2 GN (BioMérieux, France) and Bruker MALDI-ToF MS (Bruker, Germany) were not able to identify the bacteria. While in ICU on the sixth day of admission, her condition did not show improvement, and intravenous meropenem 1 g BD was initiated. Despite on meropenem, on day 8 of admission, she succumbed to the infection. Her cause of death was determined as bacteraemia with severe pneumonia and multiorgan failure. Investigation by the public health officials was unable to find any possible medium of transmission; there were no insects bite, no pets, no pests, no animal bites, no history of handling animal carcasses, no gardening, no farming, no history of eating uncooked food and the deceased’s house was well kept and has a clean water supply. In addition, she also did not have recent travel history.

**Fig. 2:**
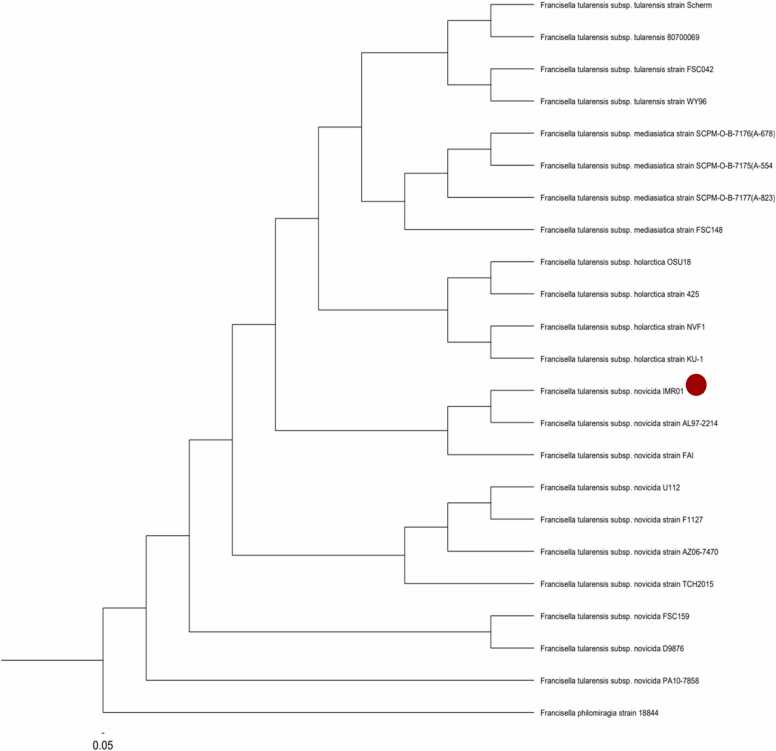
A kSNP-based whole genome phylogenetic analysis of the isolate used in this study, IMR01 (labelled with red dot) compared with other F. tularensis subspecies novicida sequences available in public databases. (Francisella tularensis subsp. tularensis strain Scherm complete genome NC_017463.1, Francisella tularensis subsp. tularensis 8070069 complete genome Genbank NZ_APKV00000000.1, Francisella tularensis subsp. holarctica OSU18, complete sequence Genbank NC_017463.1, Francisella tularensis subsp. novicida U112 chromosome complete genome Genbank NZ_CP009633.1, Francisella tularensis subsp. holarctica strain 425 chromosome complete genome Genbank NZ_CP010289.1, Francisella tularensis subsp. tularensis strain WY96 chromosome complete genome,NZ_CP012037.1, Francisella tularensis subsp. novicida PA10-7858 chromosome complete genome NZ_CP016635.1, Francisella tularensis subsp. novicida strain AL97-2214 chromosome, complete genome, NZ_CP009653.1, Francisella tularensis subsp. novicida strain AZ06-7470 chromosome complete genome NZ_CP009682.1, Francisella tularensis subsp. novicida strain TCH2015 chromosome complete genome NZ_CP021490.1, Francisella tularensis subsp. mediasiatica strain SCPM-O-B-7176(A-678) whole genome shotgun NZ_SGWO01000100.1, 1 Francisella tularensis subsp. mediasiatica strain SCPM-O-B-7177(A-823) whole genome shotgun NZ_SGWN01000100, Francisella tularensis subsp. mediasiatica strain SCPM-O-B-7175(A-554 whole genome shotgun NZ_SGWP01000100.1, Francisella tularensis subsp. holarctica strain NVF1 chromosome, complete genome NZ_AP023459.1, Francisella tularensis subsp. holarctica strain KU-1 chromosome, complete genome NZ_AP023460.1, Francisella tularensis subsp. novicida strain F1127 novicida_10, whole genome shotgun sequence NZ_JACTTJ010000009.1, Francisella tularensis subsp. novicida FSC159 FSC159_0_len801, whole genome shotgun sequence NZ_JACVLZ010000001.1, Francisella tularensis subsp. mediasiatica strain FSC148_0_len22981, whole genome shotgun sequence NZ_JACVLV010000001.1, Francisella tularensis subsp. tularensis strain FSC042 FSC042_0_len804, whole genome shotgun sequence NZ_JACVKC010000001.1, Francisella philomiragia strain 18,844 chromosome complete genome NZ_CP063138.1, Francisella tularensis subsp. tularensis 80700069 Contig01, whole genome shotgun sequence NZ_APKV01000001.1, Francisella tularensis subsp. novicida strain FAI scaffold1, whole genome shotgun sequence NZ_KN046811.1, Francisella tularensis subsp. novicida D9876 chromosome complete genome NZ_CP009607.1).

In IMR, bacterial DNA was extracted from the freshly grown isolate using MagNA Pure 96 DNA and Viral NA Small Volume Kit (Roche, Switzerland) and was subjected to a conventional polymerase chain reaction (PCR) using universal primer 27F (5′-AGAGTTTGATCCTGGCTCAG-3′) and 1492R (5′-CGGTTACCTTGTTSCGAC-3′) [10] which targets near full-length 16S rRNA gene. The bacterial 16S rRNA amplicons was sent for Sanger sequencing. The forward and reverse sequences obtained were aligned using MEGA11 software [11]. Consensus sequences were then compared with the GenBank database using BLASTN analysis which identified as *Francisella tularensis* with an identical score of 100 %. However, further discrimination among closely related species cannot be fully determined. Since this is the first time *Francisella tularensis* was identified by 16S rRNA in Malaysia, we decided to further proceed with whole genome sequencing to characterise the bacteria. The genomic library preparation DNA was performed using Oxford Nanopore Technologies (ONT) Ligation Sequencing Kit V14 (SQK-LSK114). Sequencing was performed using MinION Flow Cell (R10.4.1) on ONT GridION. The sequence reads were assembled using the EPI2ME wf-bacterial-genomes workflow that included *de novo* assembly using Flye version 2.9.1-b1780 and polished with Medaka 1.7.2 software. The assembly resulted in one circular contig with the size of 1.92 mb and the mean contig coverage of 582 ×. The identification of the bacterial species from assembly was done using Kraken 1.1.1 [12] as *Francisella tularensis* subspecies novicida. Phylogenetic analysis using kSNP 3.0 [13] demonstrated our isolate belonged in the same clade as *Francisella novicida* strain AL97-221 (Fig. 2). The assembly has been submitted to National Center for Biotechnology Information (NCBI) under accession number GenBank GCA_052692595.1.

## Discussion

This is the first reported case of human FN infection in Malaysia. Unfortunately, the case resulted in death. Identification challenges delayed the diagnosis, affecting the patient’s treatment. FN is a rare opportunistic pathogen causing infection mostly in the immunocompromised humans and/or with co-morbid [1], [2], [3], [4], [5], [7], [8], [9], [14], [15]. Whether FN should be classified as the fourth subspecies of *F. tularensis* or as a distinct species has been widely debated, with current evidence suggesting that FN is a separate species [1], [2], [14], [16]. The transmission route of FN is largely unknown, unlike FH and FT which spread through tick bites. In this case, we were unable to ascertain the mode of transmission. Most other case reports were not able to determine mode of transmission [4], [5], [8], [9], [15]. However, there were case reports with link to contaminated food and water exposure. There were no evidence of insect or animal vectors [6], [7]. FN infection presents variably, ranging from non-specific symptoms to bacteraemia, pneumonia, and skin or soft tissue infections with most recent patients having pneumonia and bacteraemia [8], [9], [14]. A compromised immune system is a common risk factor, with one exception reported in Italy, where the host is immunocompetent [8].

Identifying FN in clinical settings is challenging due to its rarity in human cases. In most cases identification can only be confirmed using molecular methods because FN is difficult to identify using conventional method. In addition, there is limited data on FN in the MALDI-ToF mass spectrometry libraries [5], [8], [9], [15]. Molecular identification for FN is currently not available in Malaysian public health hospitals. Creating main spectral libraries for FN in the Bruker MALDI-ToF mass-spectrometry could address the identification issue, as many Malaysian tertiary hospitals have Bruker's mass spectrometry biotyper instrument. Effective communication between physicians and clinical microbiologists is crucial to provide optimal empiric treatment for unidentified gram-negative coccobacilli bacteraemia. Unidentified aerobic gram-negative coccobacilli with negative oxidase and urease should be noted on the possibility of FN infection in the future as more cases of FN are being reported worldwide. Currently, there are no specific treatment guidelines established for FN infections. Majority of reported cases treatment approaches are based on *F. tularensis* infections. This is due to the rarity of FN as a human pathogen and its close resembles to *F. tularensis* genetically. Empirical treatments usually given using beta lactam antibiotics such as penicillin, ceftriaxone, or piperacillin-tazobactam. Once FN identified and susceptibility result available, targeted therapy which resembles *F. tularensis* treatment which may include doxycycline, levofloxacin, ciprofloxacin, and gentamicin are given. Treatment duration usually 14 days but depending on the severity can last from 10 to 21 days [4], [5], [6], [7], [8], [9], [15].

## Conclusion

This is the first human case of *Francisella tularensis* subspecies novicida in Malaysia. Early identification might have improved patient outcomes. New generation sequencing is valuable for identifying bacteria not detectable by common methods. Adding this bacterium to the MALDI-ToF spectral library will enable rapid future identification.

## Author Agreement Statement

We declared this manuscript has not been published in any publications before and not considered for publication elsewhere. The corresponding author is the sole contact for Editorial process and responsible for communicating with other authors about progress, submissions of revisions and final approval.

## CRediT authorship contribution statement

**Esa Habib Abdul Hakim:** Writing – review & editing, Writing – original draft, Data curation, Conceptualization. **Nazirah Samsudin:** Validation, Methodology, Investigation, Formal analysis. **Nur Fatihah Abu Bakar:** Validation, Methodology, Investigation, Formal analysis. **Letchumy Sundaraj:** Methodology, Investigation, Data curation. **Nur Asyura Nor Amdan:** Writing – review & editing, Validation, Formal analysis. **Shirley Yi Fen Hii:** Writing – review & editing, Methodology, Formal analysis. **Arusthothy Revathy:** Methodology, Investigation, Data curation. **Oon Chin Liang:** Methodology, Investigation, Data curation. **Khayri Kamel:** Writing – review & editing, Methodology, Investigation, Formal analysis. **Woo Yen Yen:** Writing – review & editing, Methodology, Data curation. **Guan Han Ling:** Writing – review & editing, Methodology, Data curation. **Rohaidah Hashim:** Writing – review & editing, Supervision, Investigation.

## Ethical approval

Written informed consent was obtained from the patient’s next of kin for publication of this case report and accompanying images. A copy of the written consent is available for review by the Editor-in-Chief of this journal on request.

## Consent

The case report approval and consent from the patient’s next of kin (the diseased), from her living brother.

## Funding source

This research did not receive any sponsorship from funding bodies in the public, commercial or not-for-profit sectors.

## Declaration of Competing Interest

All authors declare that no known competing financial interests or personal relationships could have appeared to influence the work reported in this paper.
